# The time of appearance of lung tumours in mice injected when newly born with 9,10-dimethyl-1,2-benzathracene (DMBA).

**DOI:** 10.1038/bjc.1966.18

**Published:** 1966-03

**Authors:** M. A. Walters, F. J. Roe


					
161

THE TIME OF APPEARANCE OF LUNG TUMOURS IN MICE
INJECTED WHEN NEWLY BORN WITH 9,1O-DIMETHYL-1,2-

BENZANTHRACENE (DMBA)

MARGARET A. WALTERS AND F. J. C. ROE

From the Chester Beatty Research Institute,

Institute of Cancer Research: Royal Cancer Hospital,

Fulham Road, London, S. W.3

Received for publication September 20, 1965

THE neonatal injection of carcinogenic polycyclic hydrocarbons or urethane
gives rise to a high incidence of pulmonary adenomas in many strains of mice
(Pietra, Spencer and Shubik, 1959; Pietra, Rappaport and Shubik, 1961; Kelly
and O'Gara, 1961 ; Roe, Rowson and Salaman, 1961 ; and De Benedictis et al.,
1962). An experiment has been carried out in an attempt to establish whether
the appearance of lung tumours is confined to a definite period or continued
throughout life.

MATERIALS AND METHODS

Chemical agents.-A suspension of 9,10-dimethyl-1,2-benzanthracene (DMBA)
(Roche Products Ltd.) in 3% aqueous gelatine (British Drug Houses) was prepared
by adding an acetone solution of the carcinogen to aqueous gelatine warmed to
56? C. The acetone was driven off in a stream of nitrogen. Doses of 15 ,Ig. or
30 ,ug. DMBA were contained in 0-02 ml. aqueous gelatine.

.Mice.-A breeding nucleus of " 101 " strain mice was obtained from Dr. M. F.
Lyon, Radiobiological Research Unit, Harwell, Didcot, Berks., in 1961. They
were bred by brother-sister mating and mice from the resulting colony were used
in both experiments. A cubed diet (Diet 86, Messrs. Dixon and Sons, Ware,
Herts.) and water were administered ad libitum, and the mice were housed in metal
cages. At 6 weeks of age the mice in Experiment 2 were vaccinated with sheep
lymph as a precaution against ectromelia.

Experiment 1

Newly born mice from 53 litters were injected, subcutaneously, when less than
24 hours old with 30 ,ug. DMBA. Nineteen of these litters were killed by the
mothers. From the survivors 10 mice, 5 males and 5 females picked at random,
were killed with ether 24 hours, 48 hours, 72 hours, 1 week, 2 weeks, 4 weeks,
8 weeks and 16 weeks after injection. A control group of untreated mice was
killed at 8 weeks of age. Post-mortem examination was carried out on every
animal. Before the thorax was opened a suture was tied tightly around the
trachea to prevent pulmonary collapse. The trachea, lungs, heart and thymus
were removed intact and fixed in aqueous Bouin's solution, together with the
kidneys and pieces of liver and skin. The stomachs were distended with formol
saline. Sections were stained routinely with haematoxylin and eosin. In addition,
some sections of the lungs of each mouse were stained by Gordon and Sweet's
stain for reticulin. The lungs of 2 males and 2 females in each age group were
examined in serial sections.

MARGARET A. WALTERS AND F. J. C. ROE

No neoplastic or preneoplastic changes were observed in the heart, thymus,
kidneys, liver, stomach or skin of any mouse. There were no lung adenomas in
DMBA-treated mice less than 8 weeks of age, nor in the 8 week-old untreated
controls. Quite frequently, however, groups of alveolar cells, usually situated
just beneath the pleura, were seen to be cuboidal in shape and generally similar
in appearance to those of which alveologenic adenomas are composed. Such
lesions were often associated with areas of atelactasis. Two mice (one male and
one female) killed at 8 weeks each had one small subpleural adenoma, about 0-25
mm. diameter. Another male had a nest of closely-packed alveolar cells, thought
to be an early adenoma, near the centre of one lobe. The cells partially filled the
alveolar lumen and lined the septa of several adjacent alveoli. Eight of the 10
mice killed at 16 weeks had between 1 and 4 lung adenomas each. The largest of
these was 2 mm. in diameter, but the majority were less than 1 mm.

Experiment 2

15 ,ug. DMBA in 3% aqueous gelatine was injected subcutaneously into mice
from 99 litters. Many treated mice were lost during the first week of life owing
to cannibalism. At 4 weeks the mice were weaned, sexed, numbered on the ears
and housed 4-6 to a cage. As far as possible a representative of each litter was
killed at 30, 40, 50, 60 and 70 weeks. The resulting groups consisted of 35 to 40
mice with approximately equal numbers of males and females. Post-mortem
examination was carried out on each mouse and the adenomas on the surface
of the lungs were counted. The diameter of the largest tumour was measured
and all tumours larger than 3 mm., and a sample of the smaller adenomas were
fixed and sectioned. A histological classification of lung tumours, described in an
accompanying paper (Walters, 1966) was employed. Lesions from other organs
which were definitely, or possibly, neoplastic, were also taken for microscopic
examination.

A further 30 litters were injected with aqueous gelatine alone. Groups of
about 40 mice were killed at 40 and 60 weeks and the same post-mortem procedure
was followed as for the treated mice.

Sick mice, which were killed, or mice which died during the experiment were
autopsied. Table I shows the number of deaths up to 60 weeks and the tumours
which occurred in treated and control mice. The 15 ,tg. dose of DMBA induced
a variety of tumours, including pulmonary adenomas, injection-site sarcomas,
malignant lymphomas, hepatomas, haemangiomas and skin papillomas. Sixteen
mice, including both males and females and DMBA-treated and control animals,
developed unilateral or bilateral parotid gland tumours of the type attributable
to polyoma virus infection. Rowe (1961) reported that antibodies to polyoma
virus have been detected in 0-75% of uninoculated mice in laboratory and breed-
ing colonies, though the occurrence of tumours attributable to the virus in untreated
mice is rare. Parotid tumours appeared in mice from litters which were the first
of the day's batch to be injected when a sterile needle and syringe were used.
Therefore, the possibility that the tumours were caused by the transfer of polyoma
virus from one litter, which had passive immunity, to another which had not, is
ruled out. Since there were approximately equal numbers of tumours in DMBA-
treated mice and in the aqueous gelatine controls, synergism between the virus and
DMBA, per se (Rowson et al., 1961) is unlikely. It is possible that " 101 " strain

162

APPEARANCE OF DMBA LUNG TUMOURS IN MICE

-           0

0   010   0

xo  __  U

o   I ?-       N
m~~ _      >p4o

0 _

-  ~~~0N ) C- N N

040

10   0  ~~~~~~~~~~~

o    .,                         o

0  -  0  Z~~~~~~~~~~~~~0   0 --

10 I _

00                  co o X

0~~~~~~~~~~

*qW~~~~~~~0              C3 C O O3

-     10   -   -^   -
t. XI ?X; -X; o

0  0 0~~~~0

bA~~~~~~~~~~~~~~~~~~) 1 I ^ ?

C)g C+ -9      0+- o - ?

m         0          ED0  0  0

f-4~~~-

4 _         _ _         <0

GO ~ ~ ~~ CCX- '?0 XXX   0 ;

1I d               i  I   i  ii  II  t ll   II  II

3    E n ?       O =  ?e o x d x; ? S it m

E-2  :,+     ?          ;4

163

MARGARET A. WALTERS AND F. J. C. ROE

0

:-4

0

0

C i 4

0
.0
to
0
C0

---

w w

a o ct  0

I  I   I   I

W *_ ._I

II Ig

.ee~   0

-- - -

CO.C* CO*CO

-CIO   CO -eX c

01     C
C      CG

m l10t~-  -  '4

.1C0C COO,  0

o t~~~~~~~k

-q  04 1

C . .

00 0

0o    (:4

(O

* .

CO  CCO     40

- _--      -

*0)
04

O o
r- "

01
CO

0   -
Q  _

0
C._)

Wa 4

W- - CO-
- 0

CaI 10~t-COCto

-~- : ~0COCO

01 COOCOCO

* . . . .

. . . .

CO

*-         m

4   o o oC

0)0 000)

CO O OOO1eO

-0  t ~010 t

r-  r- oo4 cs el

0)

0
CO

. . . .

0 00 0q b

_4 1q CO E

. . . .

O O C> C
* to co t-

0o

*  01
4  r4
CO

.5

oo0

~CO  3

D 00
?o  0

11

a

*  *   .     *     ~.    .   .   .

CO!~  1           bC

, *      .         .      .  .  .

~-0

10 co   r

W
0D

-               -1    -     - 1

164

0

1-
o

.as

Eq

-

. . .

o o o

* 1) CO

. * . .

0

E mX

0 0 * ^ I

-a      -     0-O-     -

Q)     ta "

E-i      om

*       .     .   .

P.

O.    g GS    q m ,-

(D?

GS

. . . .

t-

APPEARANCE OF DMBA LUNG TUMOURS IN MICE

165

mice infected with polyoma virus are more prone to develop parotid gland tumours
than are other strains.

The incidence of lung tumours and other neoplasms in treated and control mice
killed at 30, 40, 50, 60 or 70 mice is shown in Table IL. Fig. 1 and 2 show that
the mean number of lung tumours and the mean size of the largest tumour increase

a'                         Q

30     40     50     60    70            30     40     50

Weeks                                    Weeks

FIG. 1.-Number of lung tumours induced in mice by 15 pg. DMBA.

30       40      50

Weeks

60      70

60     70

FIG. 2.-Size of lung tumours induced in mice by 15 ,ug. DMBA.

0

, 2C

VI

,

0 1

E
L-

w

2  C

0

E

c

a 4
:E

E

0.4
0-

E   4

, O
0 >
E . _

_ -

a.
to

. _%

c I

a

30      40       50      60      70

Weeks

I -    I

I                       I                       I

B

4

MARGARET A. WALTERS AND F. J. C. ROE

with the length of induction period from 30 to 60 weeks. The curve flattened
from 60 to 70 weeks. There were too few survivors to include a group at 80 weeks.
Many deaths were caused by papillonephritis, a disease to which " 101 " strain
mice are prone. 50-10(% of male mice over 30 weeks were affected. The highest
incidence in females, however, was 27% at 40 weeks.

Malignant lung tumours (Class 3) (Walters, 1966) which had metastasised
throughout the lung occurred in five males, two of which were killed at 60 weeks
and three at 70 weeks. Tumours which showed intrabronchial spread but no
metastases (Class 2) were seen in one male killed at 50 weeks and two killed at
70 weeks.

DISCUSSION

Many workers have found that both the incidence and the multiplicity of
pulmonary adenomas in mice increase with the length of the induction period.
Shimkin (1940) reported that the mean number of tumours in A strain mice
injected intravenously with methylcholanthrene rose from 5 at 4 weeks to 47 at
20 weeks. For C strain mice given the same treatment a mean nodule count of
4 was recorded at 20 weeks and this rose to 40 at 32 weeks. The incidence and
multiplicity of lung adenomas in A strain mice following the injection of dibenzan-
thracene, dibenzocarbazole, benzopyrene, benzodehydrocholanthrene and diben-
zacridine rose between the 8th and 14th weeks (Andervont and Shimkin, 1940).
There was, however, only a slight further increase in the mean number of tumours
per mouse between the 14th and 20th weeks. When a comparison was made
between groups of mice which received a range of doses of methyleholanthrene,
a significant difference was found between mice kept for 8 weeks and those kept
for 13 or 18 weeks, when the dose was greater than 0 125 mg. (Shimkin and
McClelland, 1949).

Thirty per cent of newborn A strain mice injected with methylcholanthrene
developed lung adenomas by 4 weeks (Kimura and Senra, 1964). Methylcholan-
threne and dibenzanthracene gave a 5000 yield of lung adenomas at 8 weeks after
being injected into newborn albino mice (Kelly and O'Gara, 1961). Tumour
incidence rose to 96% at 24 weeks. The mean nodule count after methylcholan-
threne was 6 at 8 weeks, 26 at 16 weeks and 23 at 24 weeks: after dibenzanthracene
it was 3 at 8 weeks, 11 at 16 weeks and 24 at 24 weeks. De Benedictis et al. (1962)
found that the neonatal injection of urethane induced lung adenomas in 20% Swiss
mice at 3 weeks. The average number of tumours was 1. One hundred per cent
of the mice had tumours by 13 weeks when the mean nodule count was 5. The
mean count rose further to 17 at 30 weeks.

Strains differ in their susceptibility to the induction of pulmonary adenomas
and in the time of appearance of the earliest tumour. Kelly and O'Gara (1961),
De Benedictis et al. (1962) and Kimura and Senra (1964) describe the appearance
of lung adenomas in albino, Swiss and A strain mice only 3 or 4 weeks after the
neonatal injection of a carcinogen, but in " 101 " strain mice no tumours were
seen in mice younger than 8 weeks. Of 10 mice killed at 8 weeks, 2 had definite
adenomas and one had a lesion which was probably an early adenoma.

Lung tumour incidence was high, between 80 and 100%, in all DMBA-treated
mice killed at 30 weeks or more. The mean number of tumours per mouse and
the mean size of the largest tumour increased between the 30th and 60th week,
but the curve for the mean number of tumours per mouse levelled out between

166

APPEARANCE OF DMBA LUNG TUMOURS IN MICE                167

60 and 70 weeks. This suggests that the rate of appearance of new tumours fell
off at about 60 weeks, though the tumours already present continued to increase
in size. Unfortunately, because of small numbers due to poor survival, and
because of wide mouse to mouse variation, the figure shown (Fig. 1) for mean num-
ber of tumours at 70 weeks, is not significantly different from the number which
would have been expected if new tumours had continued to appear between the
60th and 70th weeks at the same rate as they were appearing between the 30th
and 60th weeks.

Clearly in the case of " 101 " mice injected neonatally with DMBA, there is
wide variation in the time of appearance of macroscopically visible tumours,
since new tumours were still appearing between the 50th and 60th weeks. It
remains uncertain whether there is a point in the life span of the mouse after which
lung tumours attributable to exposure to carcinogens during the neonatal period
do not occur.

SUMMARY

1. " 101 " Strain mice injected neonatally with 30 1,g. 9,10-dimethyl-1,2-
benzanthracene (DMBA) in 3%o aqueous gelatine were killed at 1 day, 2 days,
3 days, 1 week, 2 weeks, 4 weeks and 8 weeks of age. No neoplastic or preneo-
plastic lesions were seen in any organ except the lung: 2 out of 10 mice had 1
adenoma at 8 weeks and another had an early adenoma. Eight out of 10 mice
had adenomas at 16 weeks.

2. In mice injected with 15 ,ug. DMBA at birth, the average number of tumours
per mouse and the size of the largest tumour increased with age from 30 to 60
weeks. The mean nodule count at 70 weeks was similar to that at 60 weeks, but
it is not certain that the appearance of new tumours stopped or even slowed down
at 60 weeks. Tumour size continued to increase between the 60th and 70th weeks.

REFERENCES

ANDERVONT, H. B. AND SHIMKIN, M. B.-(1940). J. natn. Cancer Inst., 1, 225.

DE BENEDICTIS, G., MAIORANO, G., CHIECO-BIANCHI, L. AND FIORE-DONATI, L.-(1962)

Br. J. Cancer, 16, 686.

KELLY, M. G. AND O'GARA, R. W.-(1961) J. natn. Cancer Inst., 26, 651.
KIMURA, K. AND SENRA, Y.-(1964) J. Nara med. Ass., 15, 231.

PIETRA, G., RAPPAPORT, H. AND SHUBIK, P.-(1961) Cancer, N.Y., 14, 308.
PIETRA, G., SPENCER, K. AND SHUBIK, P.-(1959) Nature, Lond., 183, 1689.

ROE, F. J. C., ROWSON, K. E. K. AND SALAMAN, M. H.-(1961) Br. J. Cancer, 15, 515.
ROWE, W. P.-(1961) Bact. Rev., 25, 18.

ROWSON, K. E. K., ROE, F. J. C., BALL, J. K. AND SALAMAN, M. H.-(1961) Nature,

Lond., 191, 893.

SHIMKIN, M. B.-(1940) Archs Path., 29, 229.

SHIMKIN, M. B. AND MCCLELLAND, J. N.-(1949) J. natn. Cancer Inst., 10, 597.
WALTERS, M. A. (1966) Br. J. Cancer, 20, 148.

				


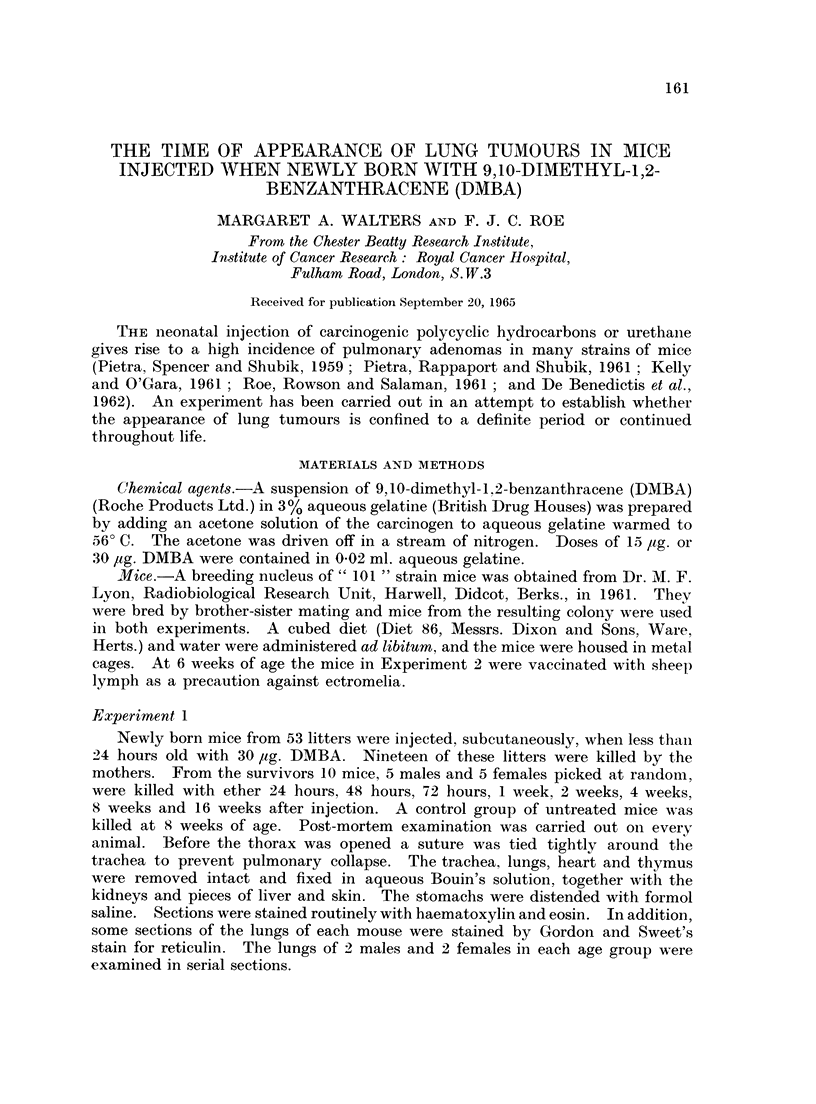

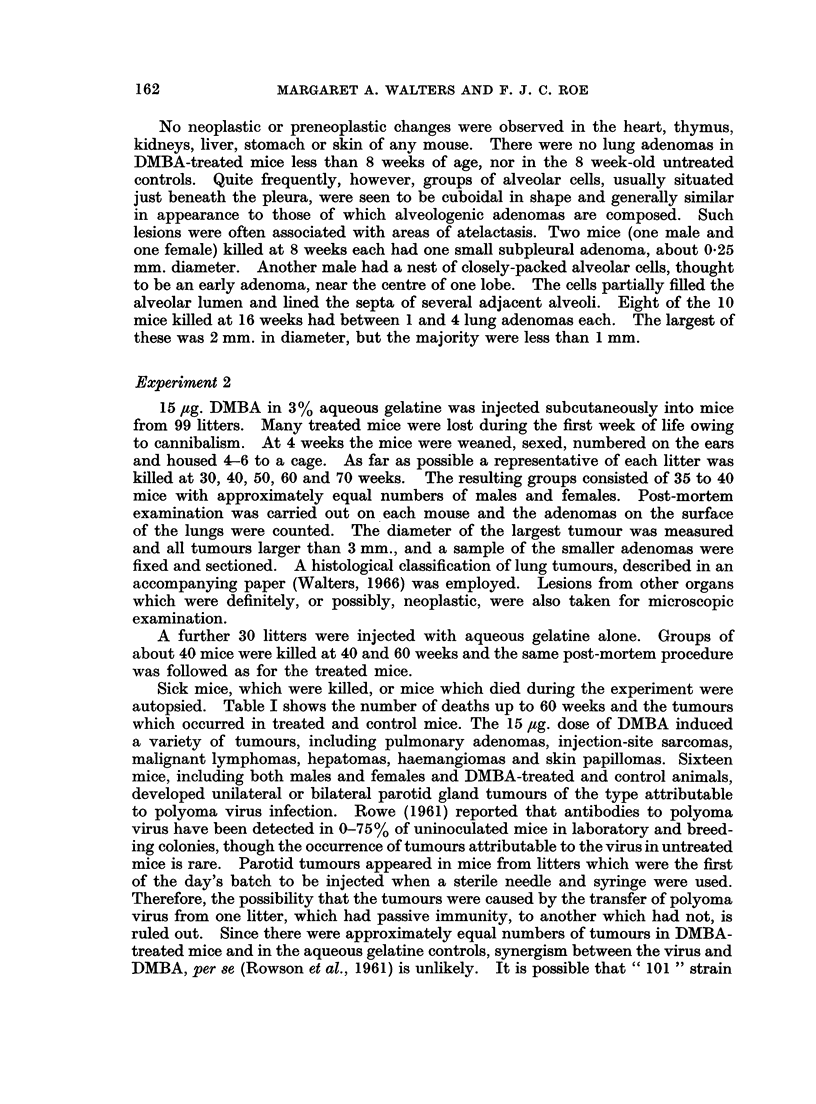

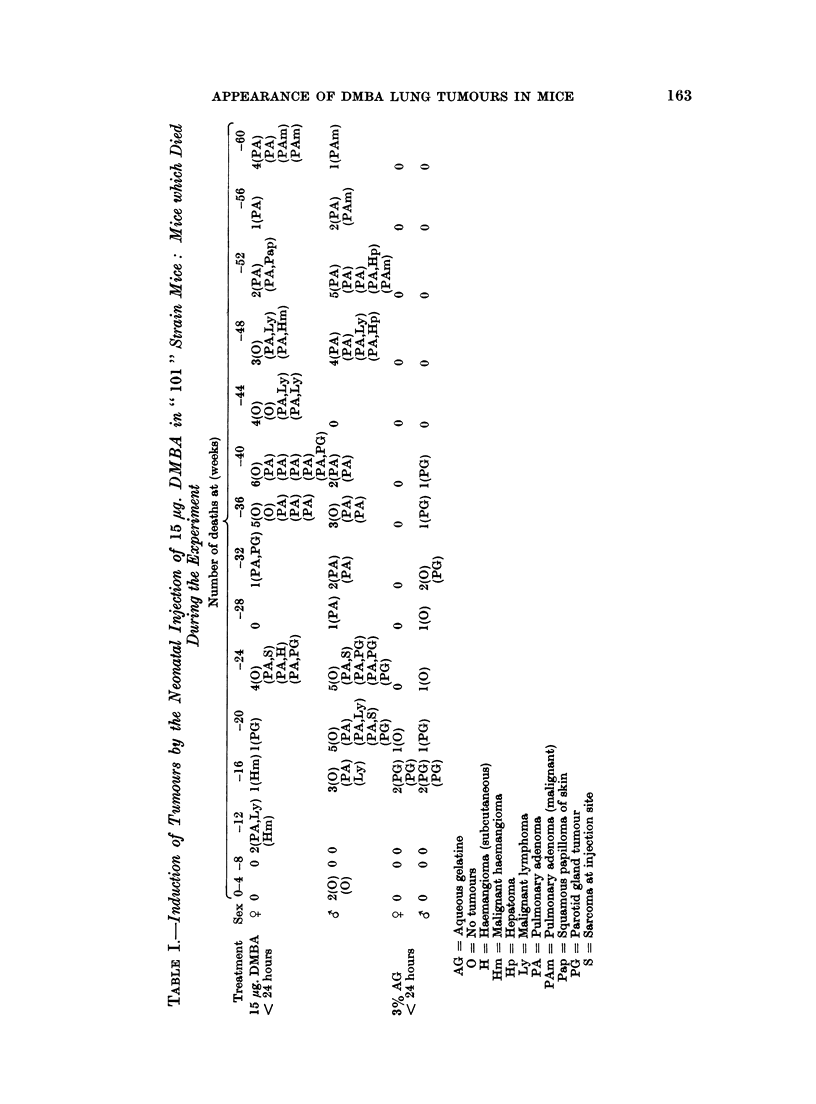

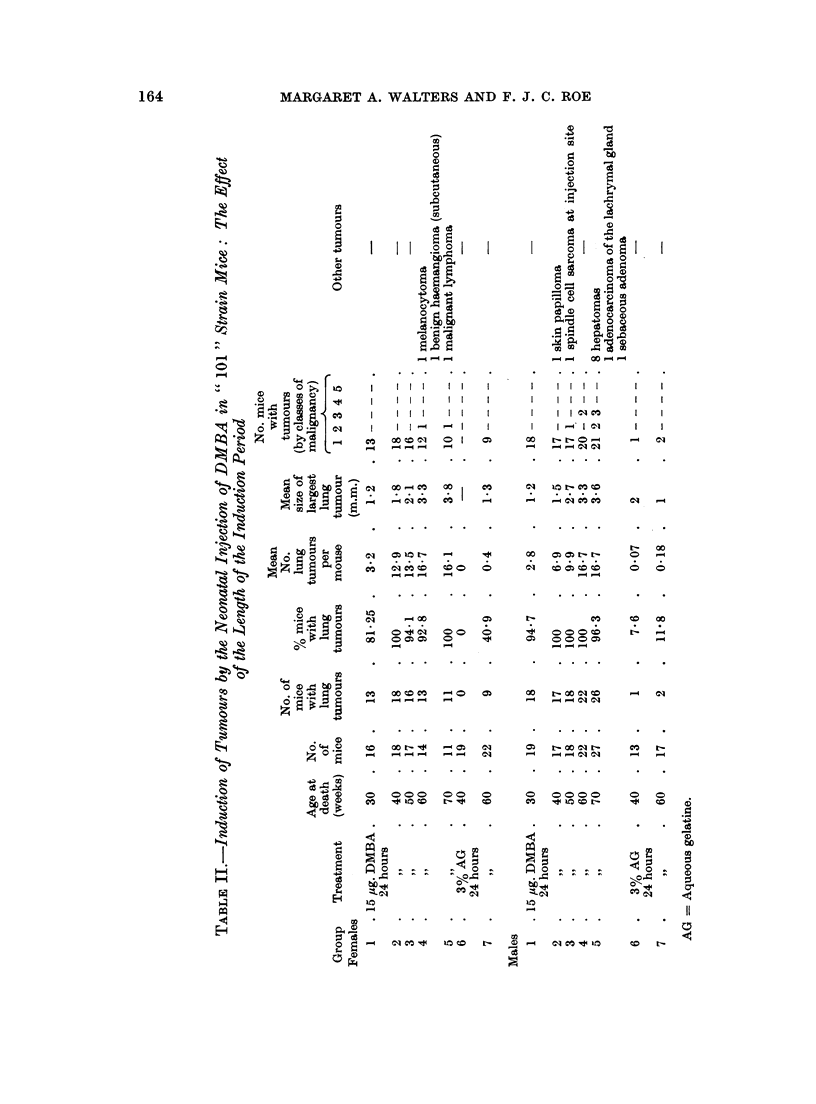

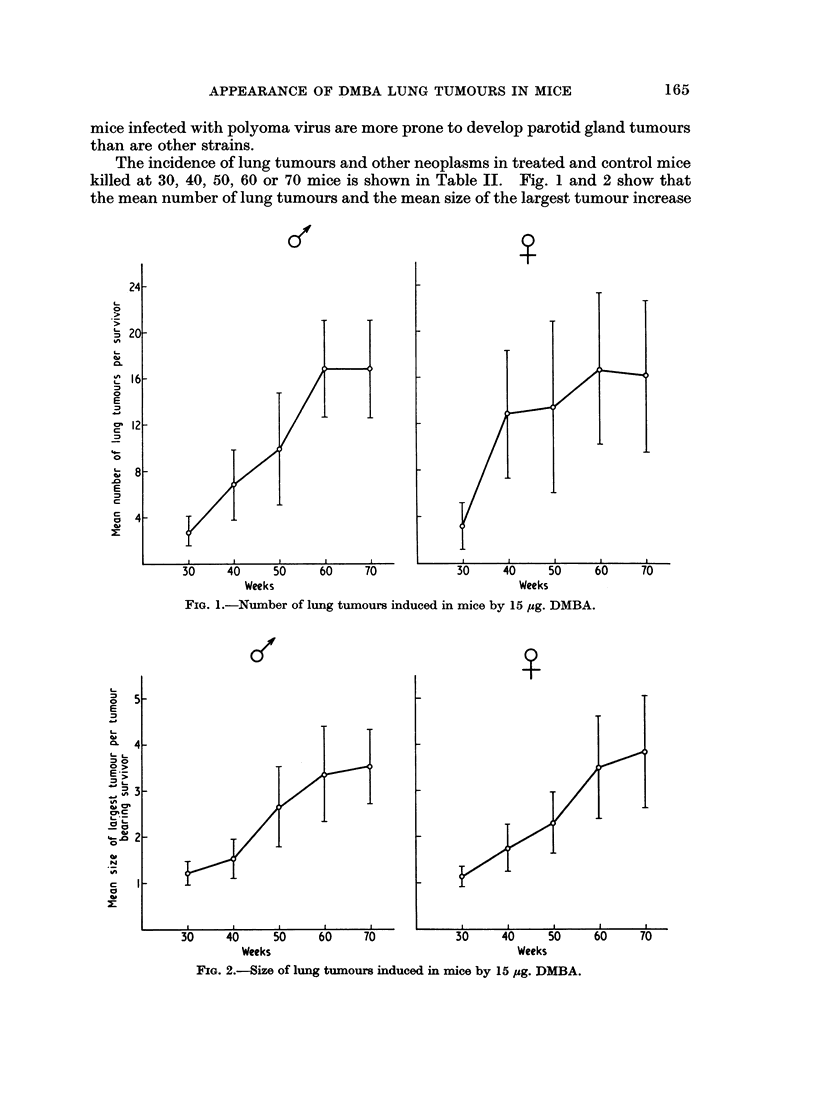

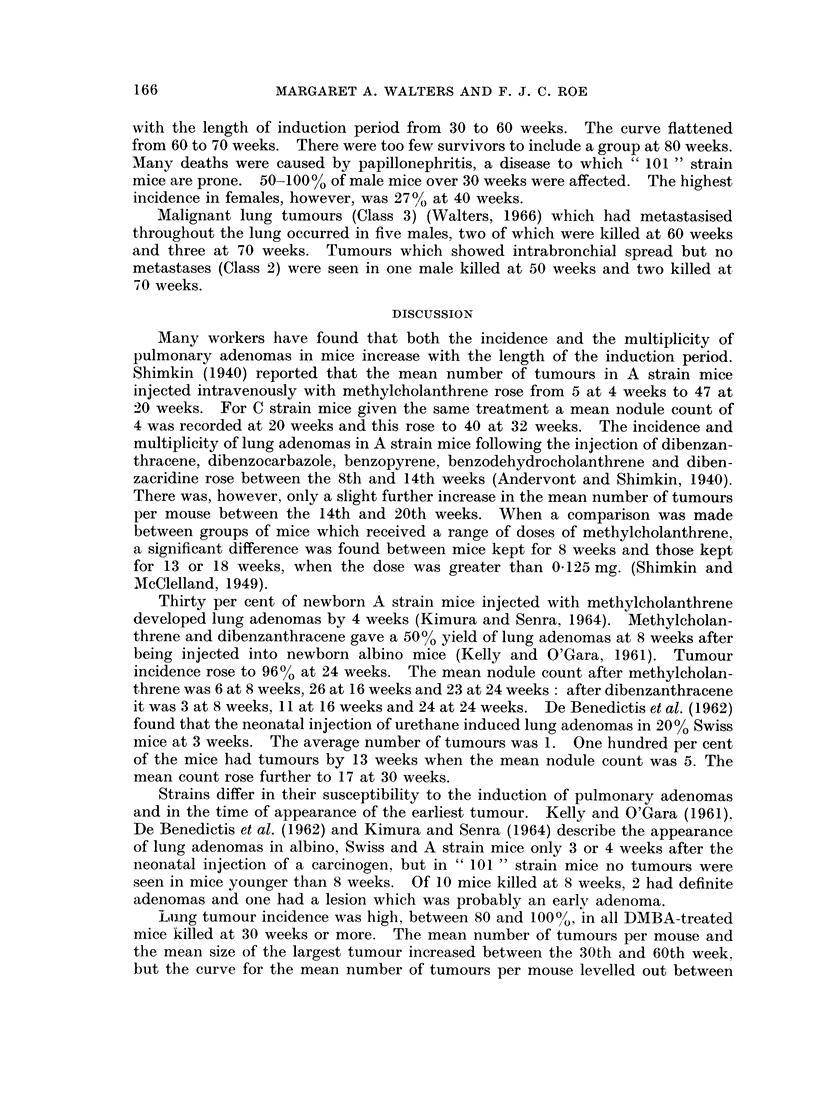

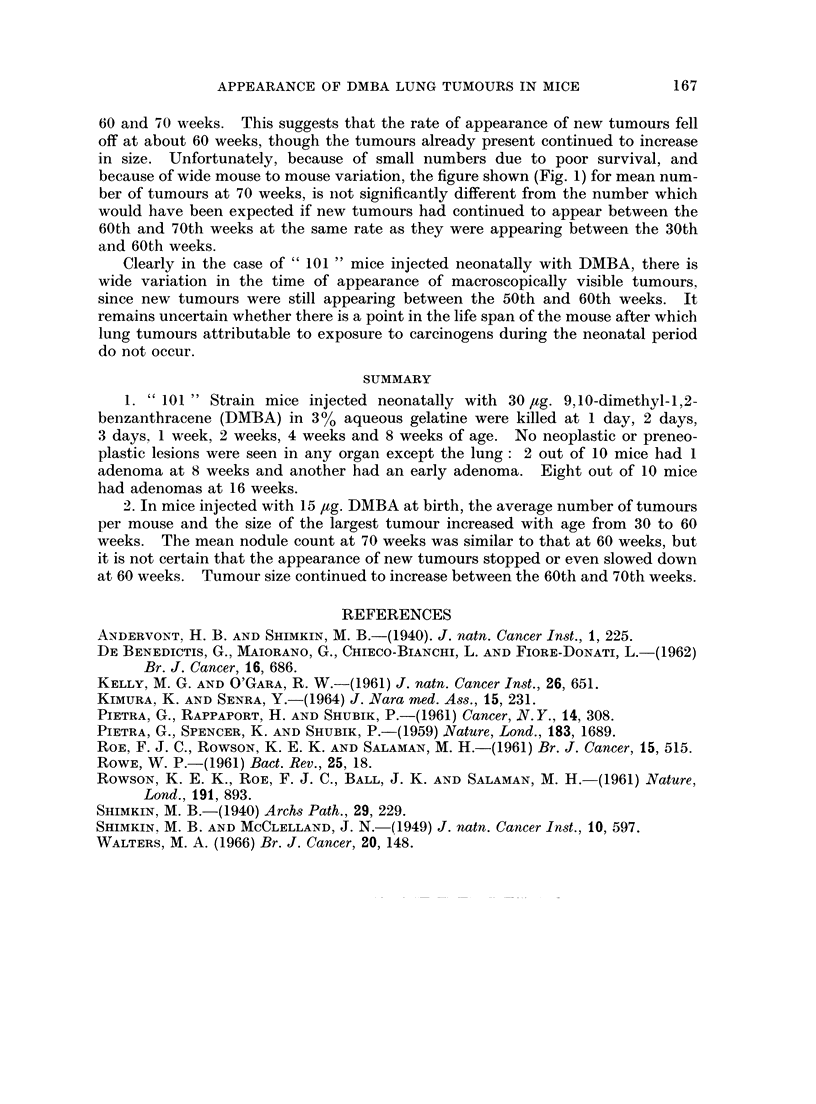

